# Early Miocene origin and cryptic diversification of South American salamanders

**DOI:** 10.1186/1471-2148-13-59

**Published:** 2013-03-04

**Authors:** Kathryn R Elmer, Ronald M Bonett, David B Wake, Stephen C Lougheed

**Affiliations:** 1 Department of Biology, Queen’s University, Kingston, Ontario, Canada; 2 Lehrstuhl für Zoologie und Evolutionsbiologie, Department of Biology, University of Konstanz, Universitätstrasse 10, Konstanz, 78457, Germany; 3 Current address: Institute of Biodiversity, Animal Health & Comparative Medicine, College of Medical, Veterinary & Life Sciences, University of Glasgow, Glasgow, UK; 4 Department of Biological Science, University of Tulsa, Tulsa, Oklahoma, USA; 5 Museum of Vertebrate Zoology and Department of Integrative Biology, University of California, Berkeley, California, 94720-3160, USA

## Abstract

**Background:**

The currently recognized species richness of South American salamanders is surprisingly low compared to North and Central America. In part, this low richness may be due to the salamanders being a recent arrival to South America. Additionally, the number of South American salamander species may be underestimated because of cryptic diversity. The aims of our present study were to infer evolutionary relationships, lineage diversity, and timing of divergence of the South American *Bolitoglossa* using mitochondrial and nuclear sequence data from specimens primarily from localities in the Andes and upper Amazon Basin. We also estimated time of colonization of South America to test whether it is consistent with arrival via the Panamanian Isthmus, or land bridge connection, at its traditionally assumed age of 3 million years.

**Results:**

Divergence time estimates suggest that *Bolitoglossa* arrived in South America from Central America by at least the Early Miocene, *ca*. 23.6 MYA (95% HPD 15.9-30.3 MYA), and subsequently diversified. South American salamanders of the genus *Bolitoglossa* show strong phylogeographic structure at fine geographic scales and deep divergences at the mitochondrial gene cytochrome *b* (*Cytb*) and high diversity at the nuclear recombination activating gene-1 (*Rag1*). Species often contain multiple genetically divergent lineages that are occasionally geographically overlapping. Single specimens from two southeastern localities in Ecuador are sister to the *equatoriana*-*peruviana* clade and genetically distinct from all other species investigated to date. Another single exemplar from the Andes of northwestern Ecuador is highly divergent from all other specimens and is sister to all newly studied samples. Nevertheless, all sampled species of South American *Bolitoglossa* are members of a single clade that is one of several constituting the subgenus *Eladinea*, one of seven subgenera in this large genus.

**Conclusions:**

The ancestors of South American salamanders likely arrived at least by the Early Miocene, well before the completion of the Late Pliocene Panamanian land bridge (widely accepted as *ca*. 3 MYA). This date is in agreement with recent, controversial, arguments that an older, perhaps short-lived, land connection may have existed between South America and present-day Panama 23–25 MYA. Since its arrival in South America, *Bolitoglossa* has diversified more extensively than previously presumed and currently includes several cryptic species within a relatively small geographic area. Rather than two upper Amazonian species currently recorded for this region, we propose that at least eight should be recognized, although these additional lineages remain to be formally described.

## Background

While the forests of South America are renowned for their diversity of frogs, including endemic clades that are both old and species rich [1-11], South American salamanders are represented only by the family Plethodontidae [12] and are considered species poor in this region. In fact, with the exception of two species of *Oedipina*, all salamanders in South America belong to the genus *Bolitoglossa* (subgenus *Eladinea*) with only 28 currently recognized species that are distributed across the entire continent [12]. Species richness is greatest in Colombia and rapidly decreases to the south and east. Less than one-ninth of all recognized neotropical salamander species are found in South America, despite its geographical area dwarfing that of Central America where the remaining diversity is concentrated [13].

One hypothesis for the lower species richness of South American salamanders is that they arrived only relatively recently via the Isthmus of Panama (or Panamanian land bridge), which is widely accepted to have closed 3 to 4 million years ago (MYA). If true, then South American plethodontids would have had less time to diversify compared to those of Central America (discussed in [13,14] and references therein; tested in [15]). However, recent geological research suggests that the Panamanian land bridge may be much older (*ca*. 23–25 MYA, [16,17] and references therein), although this remains controversial (Coates, personal communication to DBW, 8 Nov. 2011).

When, and how many times, salamanders migrated into South America has been a matter of debate: for example, Brame & Wake [18] suggested multiple migrations from the Pliocene to the Pleistocene, while Dunn [19] suggested a Late Miocene to Early Pliocene origin that would predate a 3 MY old land bridge. Estimates based on allozyme distances suggested the divergence between Central and South American species to be at least 18 MYA [20], while analyses from mitochondrial (mt) DNA sequences suggested *Bolitoglossa* were present in South America before the Pliocene land bridge closure [13,21]. Hanken & Wake [20] offered two alternative hypotheses to explain such a deep level of divergence: 1) that several deeply divergent lineages occurred in southern Central America by the Early Pliocene and each independently migrated to South America after the Late Pliocene connection was established, or 2) that *Bolitoglossa* colonized South America prior to the late Pliocene land bridge and subsequently diversified *in situ*. Parra-Olea, García-París & Wake analysed the phylogenetic relationships across *Bolitoglossa* and concluded that the South American lineages must be old (35.8-12.5 MYA, depending on the molecular clock) and predate the Isthmus of Panama [13]. This was reaffirmed with more advanced chronogram analysis, which calculated a divergence of at least 11 million years [21]. However, to date, there have been no detailed studies or discussion of South American plethodontids based on DNA sequence data and these hypotheses have never been rigorously tested.

Very little is known about South American salamanders because of the “fractal” [22], highly spatially partitioned nature of plethodontid diversity across vast underexplored areas of the upper Amazonian forests. In particular the systematics of Amazonian salamanders has proven difficult because they are generally small, have similar derived morphological features such as extensively webbed digits, reduced dentition, and subtle colour pattern differentiation [23]. Further, they have been seldom collected and thus we are missing even basic data on geographical distributions. In 1963, Brame & Wake noted that because “… biologists have always been intrigued with organisms living under novel or unusual conditions, or in unexpected regions … it is surprising that South American salamanders virtually have been ignored for so long” ([18] p. 5). Almost fifty years after this statement, there has been little progress towards quantifying the biodiversity and describing the evolutionary relationships of South American salamanders.

**Figure 1:**
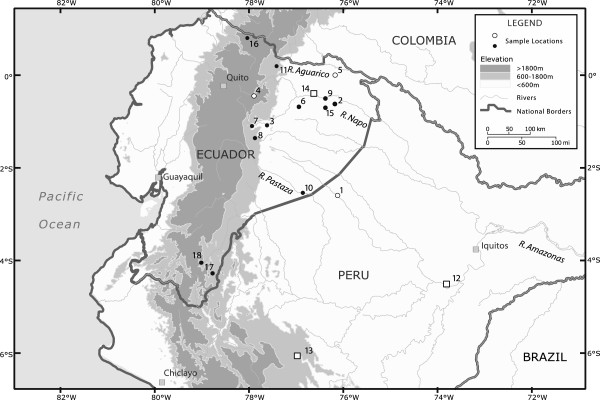
**Map of sample and type localities.** A map of sample localities of South American *Bolitoglossa* salamanders. Closed dots represent samples that were collected new for this study. Open dots represent locales for samples used in previously published research. Numbers 1 through 18 represent locality numbers from Table 1. Numbers 12, 13, and 14 (white squares) represent the type localities of *B. altamazonica*, *B. peruviana*, and *B. equatoriana*, respectively. River names are written in italics.

The aim of our present study is two-fold. First, we use divergence time estimates and ancestral area reconstruction to estimate the deepest age of an endemic South American clade and thereby infer the minimum time of colonization of South America by plethodontid salamanders. Our estimates of divergence times suggest that the salamanders arrived and diversified within South America in the Early Miocene. Furthermore, South American *Bolitoglossa* are all members of a single clade that is nested within a larger clade, the subgenus *Eladinea*, which also occurs in Costa Rica and Panama. Therefore, their colonization of South America significantly predates the widely accepted date of about 3 MYA for the Panamanian land bridge connection. Because these salamanders have low vagility and most likely reached South America by land, our results are more compatible with a much earlier land bridge connection (e.g. [16,17]). Second, we infer evolutionary relationships and the extent of genetic divergence among South American *Bolitoglossa* from the Ecuadorean Andes and upper Amazon basin (Figure 1) using mitochondrial and nuclear DNA sequence data. Our analyses reveal previously unsuspected deep genetic divergences even within local regions, documenting the presence of extensive cryptic species richness.

## Results and discussion

### *Bolitoglossa* colonization of South America

**Figure 2:**
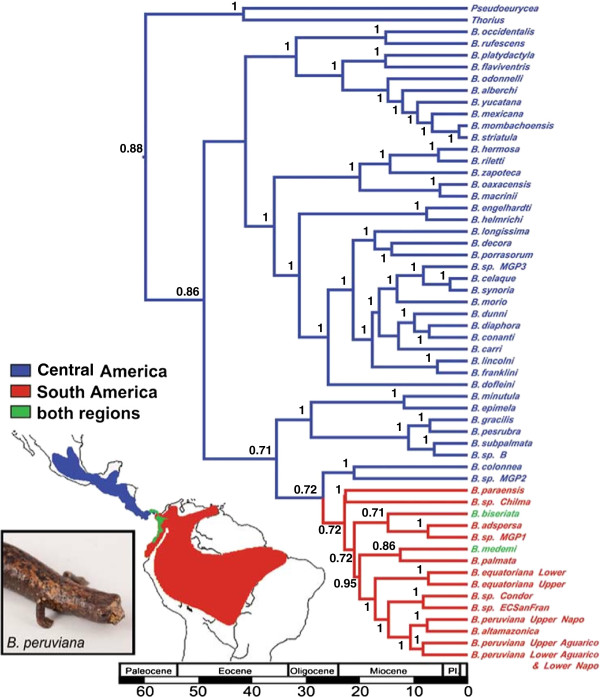
**Chronogram and ancestral area reconstruction for Central and South American *Bolitoglossa *based on *Rag1 *and *Cytb*.** Mean divergence times are based on Bayesian species tree estimation (non-bolitoglossines are not shown). Biogeographic reconstruction was calculated in Lagrange. The highest split probability for the ancestral area subtends each node, and branches are colored accordingly: Central America (blue), South America (red), and both regions (green). These analyses show a single origin of South American *Bolitoglossa* by the Early Miocene, and two recent secondary re-colonizations of Central America by species that occur in both regions.

Ancestral area reconstruction based on 56 bolitoglossine taxa strongly supported a Central American origin of *Bolitoglossa* (Figure 2). Our estimate for the deepest time of divergence for *Bolitoglossa* from throughout Central and South America is *ca*. 50 MYA (95% highest posterior density [HPD] 36.4 to 62.8 MYA, mean 50.3 MYA), and our estimate for the subgenus *Eladinea* is *ca*. 36 MYA (95% HPD 26.8 to 46.7 MYA, mean 36.5 MYA) (Figure 2, Additional file 1, Additional file 2).

**Figure 3:**
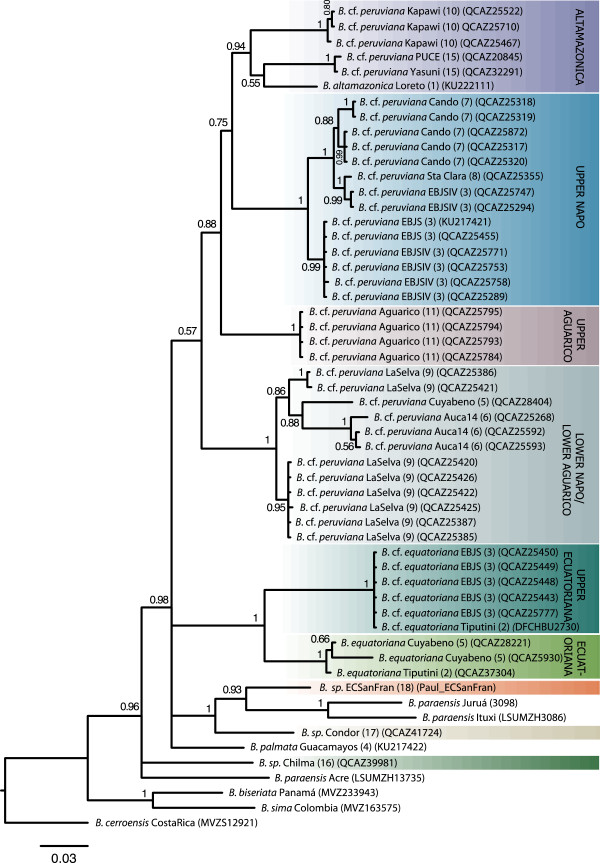
**MtDNA phylogeny focusing on South American salamanders.** The 50% majority-rule phylogenetic tree of *Cytb* inferred from Bayesian analyses. Posterior probabilities are located at nodes or above branches. Subclades referred to in the text and derived from the combined analysis are noted in vertical. See Table 1 and Figure 1 for more information on sample localities.

While we were unable to sample the South American taxa exhaustively, we found that the diverse clade of *Bolitoglossa* from the Andes and Amazon had a high likelihood of a South American origin. The origin of the entire South American clade of sampled *Bolitoglossa* was estimated to be *ca*. 23 MYA (95% HPD 15.9 to 30.3 MYA, mean 23.6 MYA; Figure 2). Even if we consider a much younger divergence time calibrations for the Plethodontidae (60 MYA), we still estimate divergence among sampled South American taxa to be at least 12.7 MYA (Additional file 2). Furthermore, the deep divergence among regionally structured clades within upper Amazonian *Bolitoglossa* — *B*. *peruviana* (*ca*. 11 MYA [95% HPD 6.9 to 15.4 MYA, mean 10.8 MYA]) and *B. equatoriana* (*ca*. 7 MYA [95% HPD 0.5 to 15.1 MYA, mean 7.4 MYA]) — also confirmed the minimally Miocene age of this radiation within South America (Figure 2, Figure 3, Additional file 1, Additional file 2). Based on these observations, we reason that the Andean and Amazonian species did not diversify north of the Panamanian land bridge and subsequently disperse into South America after its closure (one of the possibilities advanced by Hanken & Wake [20]). Rather, the lineages result from diversification in South America after colonization *ca*. 23 MYA. This is in agreement with the scenario proposed by Parra-Olea *et al*. [13], whereby the South American *Bolitoglossa* greatly predate a 3 MYA land bridge between Central and South America.

Until very recently it had generally been accepted that the South American continent was isolated from the northern land mass until the Isthmus permanently closed *ca*. 3 [24] to 4 [25] MYA. However, recent geological analyses, which are still controversial, suggest the Isthmus may have closed at least for some period of time much earlier. This involved a major narrowing of the seaway and great shifting of islands in the Early Miocene (*ca*. 23–25 MYA) [16,17] and a complete closure is now estimated to have happened by 15 MYA [26]. Clearly this would have facilitated dispersal between Central and South America. While the Pliocene ‘Great American Interchange’ between continents approximately 3 MYA considerably affected mammalian fauna [25] and some avian fauna [27,28], a growing list of biological data based on molecular phylogenetics also supports an earlier, Miocene, land connection: e.g. frogs [29,30], plants [31], procyonid mammals [32], and snakes [33]. Our finding confirms this also to be true for plethodontid salamanders. Our estimates of divergence among South American and even just Andean *Bolitoglossa* are far older than the most generally accepted dates for completion of the Panamanian land bridge *ca*. 3 MYA and instead suggest a very Early Miocene colonization of South America, which is concordant with the postulated land connection between Panama and South America on the order of 23 to 25 MYA, timing that has been advanced to explain new geological discoveries [16,17].

Since that time there have been at least two other dispersals across the Isthmus by salamanders. The two species in our analyses that occur in Colombia and Panama (*B. biseriata* and *B. medemi*) have clear South American origins but the specimens are from Central America and likely represent a more recent secondary colonization of Central America (Figure 2). Other than *Bolitoglossa*, *Oedipina* salamanders are also found in South America and these are less well known. The current taxonomy, dating from 1963 [18], recognizes two species, both of which occur in Panama. Of these, *O. complex* (type locality Gamboa, Panama) is known from two South American specimens, one from Isla Gorgona, Colombia, and the other from northwestern Ecuador. The Ecuadorean specimen has recently been re-examined (DBW, 2011) and represents an unnamed species. The second species, *O. parvipes* (type locality Cáceras, Antioquia, Colombia), is currently thought to occur in Panama, but it, too, requires a modern taxonomic revision and it is unlikely that the Panamanian and Colombian samples represent the same species. Nevertheless, whether the taxonomy is correct or not, *Oedipina* is represented by few species and in South America is found only in western Colombia and northern Ecuador. Pending study of currently unavailable samples using molecular methods, *Oedipina* may well represent a post-isthmus dispersal from its Central American origin [34].

### Phylogenetic and phylogeographic patterns within South American *Bolitoglossa*

Our phylogenetic analyses based on mtDNA document high diversity of *Bolitoglossa* lineages in the upper Amazon and Andes of Ecuador, even within a small geographic area (Figure 1, Figure 3). Additionally, there is striking geographical structure in *B. equatoriana* and *B. peruviana* often coincident with east Andean and upper Amazonian geographical features. For example, sister clades generally differ in elevation (upland vs. lowland) within and between river basins.

#### Bolitoglossa equatoriana (sensu lato) vs. B. equatoriana (sensu stricto)

**Figure 4:**
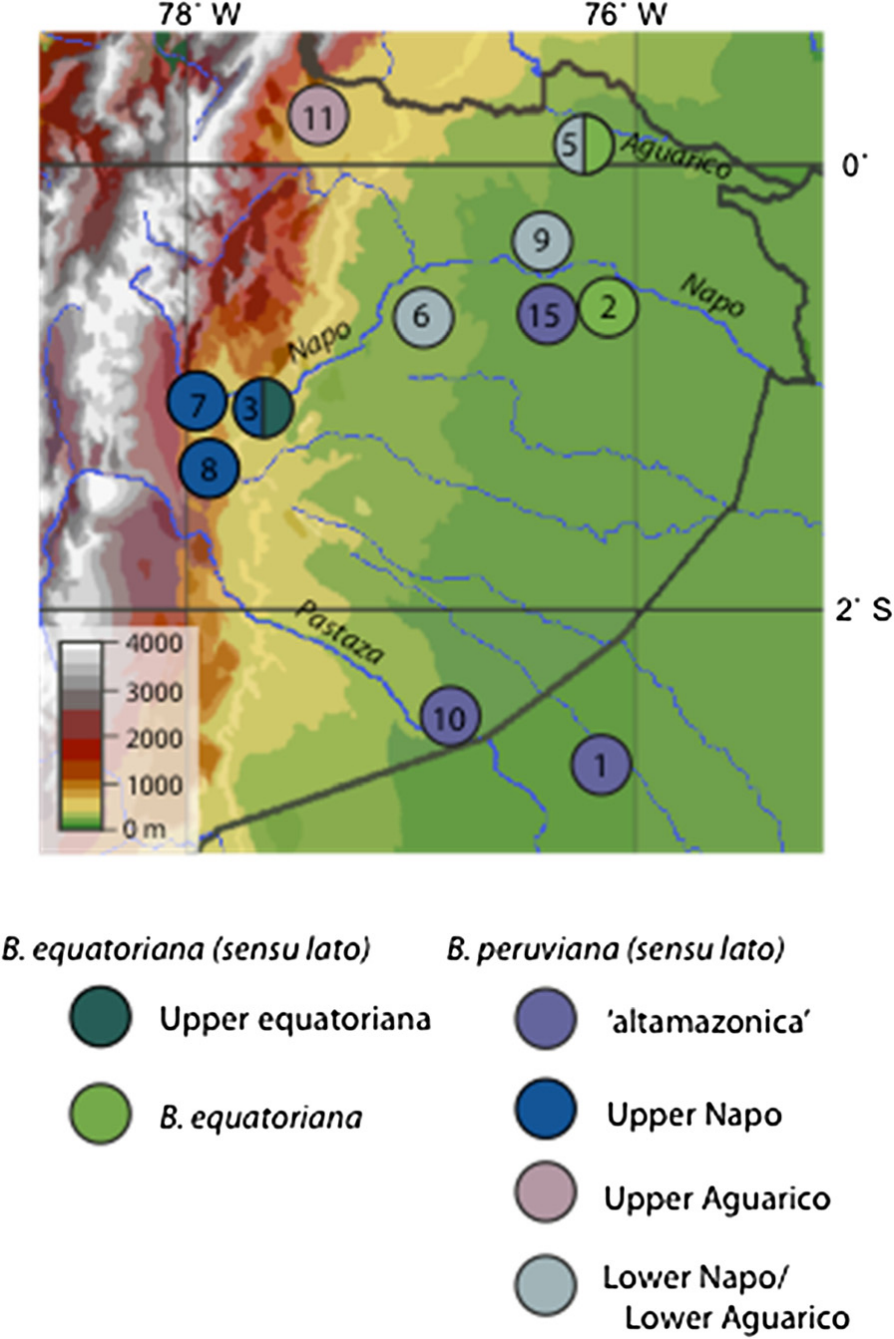
**Map of the location of the clades of *B. equatoriana *(*sensu lato*) and *B. peruviana *(*sensu lato*).** The map focuses on the upper Amazon region of Ecuador (see Figure 1). Circles represent sample localities for each of the different clades identified within *B. equatoriana* (*sensu lato*) and *B. peruviana* (*sensu lato*) from the mtDNA phylogenetic analysis (see Figure 3). Numbering follows map locality numbers (Table 1).

*Bolitoglossa equatoriana* (*sensu lato*) forms a well-supported monophyletic group (posterior probability [pp] = 1) with two divergent subclades that are separated by elevation and found in the Napo and Aguarico River Basins (Figure 3). One of these we refer to as “upper equatoriana” because it includes specimens from an upland locality on the Napo River (map locality 3; 400 metres above sea level [masl]) and a single specimen from the more lowland Tiputini locality (map locality 2; 250 masl) (Table 1, Figure 4). Its sister subclade includes samples from localities near to, and more geographically typical of, the type locality [35] (map locality 14; 260 masl) and so we consider this group to represent *B. equatoriana* (*sensu stricto*).

**Table 1:** Sample information

**Species**		**mtDNA Clade**	**Specimen number**	**Country**	**Province/Territory**	**Locality**	**Map Locality Number**	***cytb *accession**	***Rag1 *accession**
*Bolitoglossa*	*altamazonica*	altamazonica	KU222111	Peru	Loreto	1.5 km N Teniente López	1	AY526160	
*Bolitoglossa*	*biseriata*	outgroup	MVZ232943	Panama	Kuna Yala	Nusagandi	-	AY526161	KC614436
*Bolitoglossa*	*cerroensis*	outgroup	MVZ233516	Costa Rica	Cartago	Prov. Cartago	-	-	KC614459
*Bolitoglossa*	*cerroensis*	outgroup	MVZS12921	Costa Rica	San José	Cuericí 5 km E Villa Mills	-	AF199195	-
*Bolitoglossa*	cf*. equatoriana*	upper equatoriana	DFCH-BU2730	Ecuador	Napo	Estación Biológica Jatun Sacha (EBJS)	3	DQ353846	
*Bolitoglossa*	cf*. equatoriana*	upper equatoriana	QCAZ25443	Ecuador	Napo	Estación Biológica Jatun Sacha (EBJS)	3	DQ353841	
*Bolitoglossa*	cf*. equatoriana*	upper equatoriana	QCAZ25448	Ecuador	Napo	Estación Biológica Jatun Sacha (EBJS)	3	DQ353842	KC614451
*Bolitoglossa*	cf*. equatoriana*	upper equatoriana	QCAZ25449	Ecuador	Napo	Estación Biológica Jatun Sacha (EBJS)	3	DQ353843	
*Bolitoglossa*	cf*. equatoriana*	upper equatoriana	QCAZ25450	Ecuador	Napo	Estación Biológica Jatun Sacha (EBJS)	3	DQ353844	
*Bolitoglossa*	cf*. equatoriana*	upper equatoriana	QCAZ25777	Ecuador	Napo	Estación Biológica Jatun Sacha (EBJS-Inner Vision Lodge)	3	DQ353840	
*Bolitoglossa*	cf. *peruviana*	altamazonica	QCAZ20845	Ecuador	Orellana	Estación Científica Yasuní (PUCE)	15	KC614427	KC614453
*Bolitoglossa*	cf. *peruviana*	altamazonica	QCAZ25467	Ecuador	Pastaza	Kapawi Lodge	10	DQ353811	
*Bolitoglossa*	cf. *peruviana*	altamazonica	QCAZ25522	Ecuador	Pastaza	Kapawi Lodge	10	DQ353809	KC614442
*Bolitoglossa*	cf. *peruviana*	altamazonica	QCAZ25710	Ecuador	Pastaza	Kapawi Lodge	10	DQ353810	
*Bolitoglossa*	cf. *peruviana*	altamazonica	QCAZ32291	Ecuador	Orellana	Estación Científica Yasuní (PUCE)	15	KC614430	KC614455
*Bolitoglossa*	cf. *peruviana*	lower Napo/lower Aguarico	QCAZ25268	Ecuador	Orellana	Auca 14 Rd near Coca	6	DQ353830	KC614447
*Bolitoglossa*	cf. *peruviana*	lower Napo/lower Aguarico	QCAZ25385	Ecuador	Orellana	La Selva Lodge	9	DQ353835	KC614449
*Bolitoglossa*	cf. *peruviana*	lower Napo/lower Aguarico	QCAZ25386	Ecuador	Orellana	La Selva Lodge	9	DQ353833	
*Bolitoglossa*	cf. *peruviana*	lower Napo/lower Aguarico	QCAZ25387	Ecuador	Orellana	La Selva Lodge	9	DQ353836	KC614450
*Bolitoglossa*	cf. *peruviana*	lower Napo/lower Aguarico	QCAZ25420	Ecuador	Orellana	La Selva Lodge	9	DQ353838	
*Bolitoglossa*	cf. *peruviana*	lower Napo/lower Aguarico	QCAZ25421	Ecuador	Orellana	La Selva Lodge	9	DQ353832	
*Bolitoglossa*	cf. *peruviana*	lower Napo/lower Aguarico	QCAZ25422	Ecuador	Orellana	La Selva Lodge	9	DQ353834	
*Bolitoglossa*	cf. *peruviana*	lower Napo/lower Aguarico	QCAZ25425	Ecuador	Orellana	La Selva Lodge	9	DQ353839	
*Bolitoglossa*	cf. *peruviana*	lower Napo/lower Aguarico	QCAZ25426	Ecuador	Orellana	La Selva Lodge	9	DQ353837	
*Bolitoglossa*	cf. *peruviana*	lower Napo/lower Aguarico	QCAZ25592	Ecuador	Orellana	Auca 14 Rd near Coca	6	DQ353831	KC614448
*Bolitoglossa*	cf. *peruviana*	lower Napo/lower Aguarico	QCAZ25593	Ecuador	Orellana	Auca 14 Rd near Coca	6	DQ353819	KC614444
*Bolitoglossa*	cf. *peruviana*	lower Napo/lower Aguarico	QCAZ28404	Ecuador	Sucumbíos	Monte Tour, Cuyabeno	5	KC614429	KC614454
*Bolitoglossa*	cf. *peruviana*	upper Aguarico	QCAZ25784	Ecuador	Sucumbíos	Aguarico	11	DQ353813	
*Bolitoglossa*	cf. *peruviana*	upper Aguarico	QCAZ25793	Ecuador	Sucumbíos	Aguarico	11	DQ353814	
*Bolitoglossa*	cf. *peruviana*	upper Aguarico	QCAZ25794	Ecuador	Sucumbíos	Aguarico	11	DQ353815	KC614443
*Bolitoglossa*	cf. *peruviana*	upper Aguarico	QCAZ25795	Ecuador	Sucumbíos	Aguarico	11	DQ353812	
*Bolitoglossa*	cf. *peruviana*	upper Napo	KU217421	Ecuador	Napo	Estación Biológica Jatun Sacha (EBJS)	3	AY526170	
*Bolitoglossa*	cf. *peruviana*	upper Napo	QCAZ25289	Ecuador	Napo	Estación Biológica Jatun Sacha (EBJS-Inner Vision Lodge)	3	DQ353826	
*Bolitoglossa*	cf. *peruviana*	upper Napo	QCAZ25294	Ecuador	Napo	Estación Biológica Jatun Sacha (EBJS-Inner Vision Lodge)	3	DQ353816	
*Bolitoglossa*	cf. *peruviana*	upper Napo	QCAZ25317	Ecuador	Napo	Cando	7	DQ353822	
*Bolitoglossa*	cf. *peruviana*	upper Napo	QCAZ25318	Ecuador	Napo	Cando	7	DQ353824	
*Bolitoglossa*	cf. *peruviana*	upper Napo	QCAZ25319	Ecuador	Napo	Cando	7	DQ353823	
*Bolitoglossa*	cf. *peruviana*	upper Napo	QCAZ25320	Ecuador	Napo	Cando	7	DQ353821	KC614445
*Bolitoglossa*	cf. *peruviana*	upper Napo	QCAZ25355	Ecuador	Pastaza	Sta Clara (finca de Tapia)	8	DQ353818	
*Bolitoglossa*	cf. *peruviana*	upper Napo	QCAZ25455	Ecuador	Napo	Estación Biológica Jatun Sacha (EBJS)	3	DQ353829	
*Bolitoglossa*	cf. *peruviana*	upper Napo	QCAZ25747	Ecuador	Napo	Estación Biológica Jatun Sacha (EBJS-Inner Vision Lodge)	3	DQ353817	
*Bolitoglossa*	cf. *peruviana*	upper Napo	QCAZ25753	Ecuador	Napo	Estación Biológica Jatun Sacha (EBJS-Inner Vision Lodge)	3	DQ353827	KC614446
*Bolitoglossa*	cf. *peruviana*	upper Napo	QCAZ25758	Ecuador	Napo	Estación Biológica Jatun Sacha (EBJS-Inner Vision Lodge)	3	DQ353825	
*Bolitoglossa*	cf. *peruviana*	upper Napo	QCAZ25771	Ecuador	Napo	Estación Biológica Jatun Sacha (EBJS-Inner Vision Lodge)	3	DQ353828	
*Bolitoglossa*	cf. *peruviana*	upper Napo	QCAZ25872	Ecuador	Napo	Cando (Serena North side)	7	DQ353820	
*Bolitoglossa*	*equatoriana*	equatoriana	QCAZ05930 (= LSUMZ-H12838)	Ecuador	Sucumbíos	Cuyabeno Reserve	5	AY526169 Previously reported as *B. peruviana* (Parra-Olea et al 2004)	
*Bolitoglossa*	*equatoriana*	equatoriana	QCAZ28221	Ecuador	Sucumbíos	Pto Bolivar (Cuyabeno)	5	KC614428	
*Bolitoglossa*	*equatoriana*	equatoriana	QCAZ37304	Ecuador	Orellana	Tiputini Reserve	2	DQ353845	KC614452
*Bolitoglossa*	*palmata*		KU217422	Ecuador	Napo	Guacamayos 31 km de Baeza	4	AY526164	
*Bolitoglossa*	*paraensis* (Acre)		LSUMZH-13735	Brazil	Acre	5 km N Porto Walter	-	AY526168	
*Bolitoglossa*	*paraensis* (Ituxi)		LSUMZH-3086	Brazil	Amazonas	Rio Ituxi at Madeireira Scheffer	-	AY526167	
*Bolitoglossa*	*paraensis* (Jurua)		3098	Brazil	Amazonas	Río Jurua INPA	-	AY526166	
*Bolitoglossa*	*sima*		MVZ163575	Colombia		Valle de Cauca	-	AY526172	
*Bolitoglossa*	*sp.* (Chilma)		QCAZ39981	Ecuador	Carchi	Chilma Bajo	16	KC614431	KC614456
*Bolitoglossa*	*sp.* (Condor)		QCAZ41724	Ecuador	Zamora-Chinchipe	Cordillera del Cóndor, near San Miguel de Las Orquídeas	17	KC614432	KC614456
*Bolitoglossa*	*sp.* (ECSanFran)		JCS 019	Ecuador	Zamora-Chinchipe	Estación Científica San Francisco	18	KC699921	KC699927

Taxon, voucher specimen museum catalogue number, geographical locality (country, province/territory and locality), locality number from Figure 1, and GenBank accession numbers. Institutional abbreviations are as listed in [36] with the following additions: BU = Boston University (USA), DFCH-USFQ = Diego Francisco Cisneros-Heredia at Universidad San Francisco de Quito (Ecuador), FHGO = Fundación Herpetológica Gustavo Orcés (Ecuador), QCAZ = Museo de Zoología Pontificia Universidad Católica (Ecuador), JCS = field collection Juan Carlos Sánchez.

Average genetic divergence between these two subclades of *B. equatoriana* (*sensu lato*) is 11.7% for *Cytb* (Table 2) and 0.8% for *Rag1* (Table 3). Bayesian estimates of divergence times average *ca*. 7.4 MYA (Figure 2).

**Table 2:** **Average within- and among-clade *Cytb *K2P corrected distances for the Ecuadorean upper Amazon salamanders**

	**Clade**	**1**	**2**	**3**	**4**	**5**	**6**
1	*peruviana*-lower Napo/lower Agua Rico	**0.033±0.005**	±0.013	±0.011	±0.013	±0.017	±0.014
2	*peruviana*-upper Napo	0.119	**0.021±0.004**	±0.011	±0.012	±0.016	±0.017
3	*peruviana*-altamazonica	0.110	0.108	**0.062±0.008**	±0.010	±0.017	±0.015
4	*peruviana*- Agua Rico	0.111	0.095	0.095	**0**	±0.018	±0.017
5	upper equatoriana	0.161	0.156	0.184	0.163	**0**	±0.014
6	*equatoriana* Brame & Wake 1972	0.136	0.158	0.161	0.153	0.117	**0.022±0.005**

Mean p-distances between subclades are written on the lower diagonal ± standard error on the upper matrix; mean intraclade p-distances ± standard error lie on the diagonal (bold type).

**Table 3:** **Average within- and among-clade *Rag1 *uncorrected p-distances for the Ecuadorean upper Amazon salamanders**

	**Clade**	**1**	**2**	**3**	**4**	**5**	**6**
1	*peruviana*-lower Napo/lower Agua Rico	**0.013±0.003**	±0.003	±0.003	±0.005	±0.005	±0.003
2	*peruviana*-upper Napo	0.012	**0.006±0.003**	±0.002	±0.006	±0.005	±0.004
3	*peruviana*-altamazonica	0.015	0.011	**0.016±0.003**	±0.005	±0.005	±0.003
4	*peruviana*- Agua Rico	0.025	0.027	0.028	**nc**	±0.007	0.006
5	upper equatoriana	0.023	0.022	0.024	0.033	**nc**	±0.003
6	*equatoriana* Brame & Wake 1972	0.015	0.013	0.016	0.26	0.008	**nc**

Mean p-distances between subclades are written on the lower diagonal ± standard error on the upper matrix; mean intraclade p-distances ± standard error lie on the diagonal (bold type).

#### Bolitoglossa peruviana diversity

Within *B. peruviana* (*sensu lato*) four major subclades are broadly distributed by elevation and river basin (Figure 3, Figure 4). We informally identify these subclades as follows: altamazonica, Upper Napo, Upper Aguarico, and Lower Napo/Lower Aguarico. The altamazonica subclade spans the greatest latitude: from the lower Napo to the lower Pastaza River Basins (map localities 1, 10, 15). This subclade, which includes the most lowland representative in our study (*B. altamazonica* Loreto), is not the sister taxon of the other Amazonian basin lowland species from Brazil, tentatively assigned to *B. paraensis*. Instead, the specimens assigned to *B. paraensis* (from Ituxi and Juruá) are more closely related to the southeastern Ecuadorean specimens from higher elevations (localities 17 and 18). The upper Napo subclade occurs in multiple localities in the vicinity of the headwaters of the Napo River (localities 3, 7 and 8). Although the southeastward distribution of this subclade remains to be determined, it is replaced to the north at the headwaters of the Aguarico River (locality 11; 610 masl) with the divergent sister subclade upper Aguarico. Specimens in the subclade lower Napo/lower Aguarico were found to span the lower Napo and lower Aguarico river basins (localities 5, 6, 9). Additional sampling is required to determine the eastward distribution of this subclade.

The four major subclades of *B. peruviana* (*sensu lato*) are highly divergent: from 9.5 to 11.9% at *Cytb* (Table 2) and from 1.1 to 2.8% at *Rag1* (Table 3; see Additional files 3 and 4 for inter-individual distances). Divergence time estimates among populations that have been assigned to *B. peruviana* average *ca*. 10.8 MYA (Figure 2).

Given the highly localized nature of genetic differentiation in what has been considered to be *B. peruviana*, we think that none of the Ecuadorean samples should be assigned to that taxon. Instead, *B. peruviana* should be considered to be a Peruvian endemic that so far is known only from the unique holotype (locality 12 in Figure 1). Further sampling, especially in this area of southern Ecuador and northern Peru, is required to further resolve phylogenetic relationships.

Our mtDNA phylogeny contains more extensive sampling of upper Amazonian salamanders than the Parra-Olea et al. [13] mtDNA phylogeny, which contained samples across the large, geographically widespread genus *Bolitoglossa*. Parra-Olea et al. [13] considered their specimen from Cuyabeno (locality 5) to be *B. peruviana* (QCAZ05930, was LSUMZ-H12838 in [13]). We find this specimen instead to be a member of the *B. equatoriana* (*sensu stricto*) clade and was likely previously misidentified. We do not have a *Rag1* sequence for this specimen.

#### Ecuadorean highland Bolitoglossa spp

Taking a broader geographical perspective on our mtDNA phylogenetic hypothesis, the high Andean specimen from northwestern Ecuador (*B*. sp. Chilma) is recovered as sister to the rest of our east Andean and Amazonian specimens (pp = 0.96; Figure 3). Its relationship with the lowland Amazonian species *B. paraensis* (Acre) is undefined. Specimens from the high altitude eastern slopes of the southern Andes of Ecuador (*B*. sp. ECSanFran, *B*. sp. Condor, *B. palmata*; localities 4, 18, 19; 1800–2000 masl) are all, with varying resolution and including the extreme lowland Brazilian *B. paraensis*, sister to the upper Amazonian species *B. peruviana* and *B. equatoriana*.

### Nuclear phylogeny

**Figure 5:**
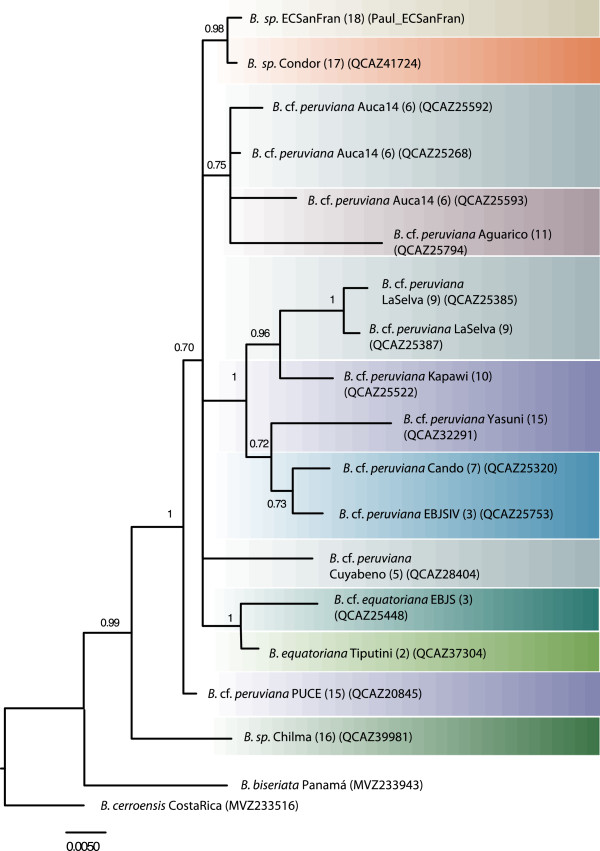
***Rag1 *phylogeny focusing on South American salamanders.** The 50% majority-rule phylogenetic tree of *Rag1* inferred from Bayesian analyses. Posterior probabilities are located at nodes or above branches. Clades referred to in the text and derived from the combined analysis are noted in vertical. See Table 1 and Figure 1 for more information on sample localities.

Phylogenetic analysis of *Rag1* alone (19 specimens) reveals weak phylogeographic structure (Figure 5), perhaps due to incomplete lineage sorting and the longer coalescence times of slowly evolving nuclear genes. As in the mtDNA phylogeny, the high Andean specimen from northwestern Ecuador (*B*. sp. Chilma) (pp = 0.99) is sister to the rest of our east Andean and Amazonian specimens. The remaining internal relationships are less defined. The combined analysis (*Rag1* and *Cytb* from 19 specimens; Additional file 5) is intermediate in resolution between the single gene analyses.

### Patterns of diversity in the upper Amazon

Similar high diversity has been found previously in Amazonian frogs, with morphological, bioacoustic, and molecular data across ecologically and evolutionarily diverse clades, including the pattern of elevation and latitude in discerning upper Amazonian clades. Specifically, the geographical distribution of mtDNA clades in *Bolitoglossa* is approximately coincident with those identified for the terrestrial leaf litter frog *Pristimantis* “*ockendeni*” [5] (now split into three species [37]) and *Engystomops* frogs [38]. The major phylogeographic divergence within northwestern Amazonian terrestrial frogs of the species *Engystomops* (=*Physalaemus*) *petersi* is also altitudinal in the Napo Basin [39-41], although elevation was not an important overall biogeographic signal at broader scales in that species [39]. Given the paucity of data on the phylogenetic diversity and fine-scale phylogeographic structure of upper Amazonian vertebrates in this region, the extent to which elevation has promoted speciation and maintains species identity (as has been inferred for salamanders [42-44] and other taxonomic groups in North [45] and Central America [46]) requires further and detailed analyses combining genetic, phenotypic, and geographical information.

### *Bolitoglossa* species richness and relevance for conservation

The high divergence and pronounced geographic structuring among subclades of these salamanders support our view that additional species of *Bolitoglossa* should be recognized in the upper Amazon region of Ecuador; more species exist than were previously recognized based on existing morphological descriptions and field surveys [18,23,35,47-50]. We propose that at least six upper Amazonian species (four within *B. peruviana sensu lato*, two within *B. equatoriana sensu lato*) be recognized, rather than the previously designated two (*B. equatoriana* and *B. peruviana*). Additionally, perhaps two more species should be named from higher elevations to the south (*B*. sp. Condor and *B*. sp. ECSanFran). It is doubtful that any Ecuadorean salamander are conspecific with topotypic *B. peruviana* because the geographic distance from the most lowland Ecuadorean sample to the *B. peruviana* type locality is greater than the ranges of the three or four phylogenetic species (i.e. clades, as described here) within Ecuador. The situation with *B. equatoriana* is also difficult, although the geographic proximity of the *B. equatoriana* type locality to specimens of the “lower equatoriana” clade suggests that this group is *B. equatoriana sensu stricto*. From northern Ecuador, in the westward drainage of the Andes Mountains, we have a single exemplar of a currently unnamed highly divergent species (*B*. sp. Chilma). Our study reveals that many taxonomic problems remain to be resolved for these morphologically similar species and work on this topic is currently underway (Wake et al. in prep.). Further alpha-taxonomic work and extensive sampling from the upper Amazon and Andes is much needed, though hampered by difficulty in collecting these rare amphibians from remote regions.

High genetic differentiation within “species” of Neotropical salamanders is not unusual in the few molecular studies on these to date (e.g. [51-54], Rovito et al. in prep). Garcia-Paris et al. [55] found genetic structuring at fine geographical scales for salamanders in continuous habitat in montane Costa Rica. In a study of allozyme variation, Hanken & Wake [20] reported that South American *Bolitoglossa* displayed intra-site heterogeneity, high differentiation within species-groups, and diversity levels comparable to among species of different genera in North America. Studies of Neotropical frog taxa have also found mtDNA differentiation to be particularly high among populations, sometimes also suggestive of distinct species [5,8,9,56,57], though not always [11]. The low vagility, high philopatry, small home ranges, and non-migratory life history of direct-developing salamanders and terrestrial frogs may promote high levels of local differentiation as a result of restricted gene flow [58], ultimately producing high speciation rates.

Our survey of salamanders from upper Amazon drainage of Ecuador indicates that upper Amazonian salamander species’ distributions are much smaller than previously assumed. Accordingly, a revision of current conservation assessments may be required. The IUCN Red List considers all salamander species east of the Andes as data deficient with regard to their risk assessment criteria (http://www.iucnredlist.org/). The Amazonian species are all thought to have very wide geographical distributions (e.g. *B. altamazonica* is purported to have a distribution including Colombia, Brazil, Venezuela, Ecuador, Peru, and Bolivia [12]) and therefore large population sizes are assumed; such widespread species have accordingly been considered of “Least Concern” [59-61]. Our findings suggest that wide geographical distributions are unlikely and species must be reassessed to take into account the possibility of smaller geographic distributions and concomitantly smaller population sizes, which might place these species at greater risk due to localized environmental degradation. Results to date imply that the salamander species richness in the Andes and upper Amazon has been substantially underestimated.

## Conclusions

Minimal estimates for the timing of diversification of the South American salamanders of the genus *Bolitoglossa*, based on our studies of eastern Ecuadorean species, indicate that plethodontids colonized South America *ca*. 23 MYA. These findings are consistent with an Early Miocene, rather than Pliocene, initial closure of the Isthmus of Panama. Within South America, *Bolitoglossa* have considerable genetic diversity at nuclear and mitochondrial loci. There is a high cryptic diversity of distinct lineages, even within a relatively small geographic area of the Andes and upper Amazon of Ecuador. Our molecular phylogenetic findings suggest that salamander species richness in South America may be seriously underestimated, in large part because of extensive morphological similarity and lack of sampling. This implies that many more species of *Bolitoglossa* should be recognized in the upper Amazon and Andes.

## Methods

### Taxon sampling

Specimens were collected opportunistically from tropical forest localities in Ecuador (Figure 1). Individuals were euthanized using chloretone or MS-222 with approved protocols, liver tissue removed, then fixed with 10% formalin and stored in 70% ethanol. Tissue samples were stored in pure ethanol until DNA analysis. Voucher specimens are deposited at the Museo de Zoología, Pontificia Universidad Católica del Ecuador (QCAZ) and Fundación Herpetológica Gustavo Orcés collections (FHGO). Sample localities, museum voucher numbers, and Genbank accession numbers are listed in Table 1. For upper Amazonian localities, specimens were assigned to *B. equatoriana* Brame & Wake, 1972 [35] or *B. peruviana* (Boulenger, 1883; see [18]) based on body size, shape and colouration.

### DNA extraction, amplification, and sequencing

Total genomic DNA was extracted from ethanol-preserved liver tissue using a Qiagen DNEasy kit (Qiagen, Inc.) following the manufacturer’s protocol, eluted in Buffer AE, and stored at -20°C.

A 790 base pair fragment of the mitochondrial gene cytochrome *b* (*Cytb*) was amplified by polymerase chain reaction (PCR) in an Applied Biosystems 2700 or 9700 thermocycler with parameters following [13]. PCR reactions used 0.3 μM of each primer (MVZ15L and MVZ16H, [62]), PCR enhancing buffer (2.5 mM MgCl2, 10 mM Tris pH 8.4, 50 mM KCl, 0.02 mg bovine serum albumin, 0.01% gelatin), 0.3 mM of each dNTP, 0.625 units of taq DNA polymerase (Fermentas), and approximately 1 to 3 ng DNA per 50 μL reaction. All amplifications included a negative control.

For representative individuals from major mitochondrial clades (see below), an 805 bp portion of the nuclear gene *Rag1* was amplified using 0.2 μM of each of the newly designed primers Rag1BolitoF (5^′^-CTT GAA CTA GGG GGC ATA CTC AGA AC-3^′^) and Rag1BolitoR (5^′^-TGC CTG GCA TTC ATT TTC CGG AAA CG-3^′^). Typical PCR conditions were as follows: 5 μL of 10X Promega PCR buffer, 1 mM MgCl2, 0.4 μM of each dNTP, 0.5 to 1 μL of a variety of taq polymerases, and approximately 2 to 4 ng of DNA per 50 μL PCR reaction. Again all amplifications included a negative control.

PCR products of the correct molecular weight were excised after electrophoresis on an agarose gel and purified using Qiagen Gel Extraction kits (Qiagen, Inc.). Big Dye (Applied Biosystems Inc.) was used for cycle sequencing reactions, which were then sequenced on an Applied Biosystems 3730 capillary sequencer. All *Cytb* samples were sequenced in one direction using the MZV15L primer and a subset of five samples was sequenced in both directions. Detailed visual comparison of forward and reverse sequences in the overlapping regions showed no discordance in DNA sequence. All *Rag1* samples were sequenced in both directions.

*Bolitoglossa* sequences were assembled and aligned in MacClade version 4.07 [63]. Nucleotide sequences were compared to protein sequences using BLASTX (http://www.ncbi.nlm.nih.gov/) to infer reading frames. Additional sequences were obtained from GenBank (Table 1).

### Divergence time estimates

We estimated a *Bolitoglossa* species tree and divergence times via Bayesian methods using the *BEAST function in the program BEAST v. 1.6.2 [64]. The species tree was based on 805 bp of Rag1 for 31 taxa and 522 bp of *Cytb* for 110 taxa (Additional file 6). This allowed us to incorporate other previously published *Cytb* sequences for other South American *Bolitoglossa* and other major clades of *Bolitoglossa* from Central America [13]. The final data set included 60 “species” (tips), with representatives of the major lineages of *B. equatoriana* and *B. peruviana*, and two non-*Bolitoglossa* bolitoglossines (*Pseudoeurycea* and *Thorius*). We also included representatives of other plethodontids (*Aneides*, *Ensatina*, *Plethodon*, and *Desmognathus*) in order to take advantage of the crown group of plethodontids as a basal calibration point for our analyses. Missing data were filled in for taxa in which we only had one of the two genes.

This sequence data matrix was partitioned by gene and codon position using substitution and site heterogeneity models determined in Modeltest (Additional file 7, see Phylogenetic Analysis section). This analysis was based on an uncorrelated lognormal molecular clock and a Yule speciation prior across the tree. We calibrated the tree with a normally distributed prior basal calibration prior for the crown group Plethodontidae. A wide range of dates has been proposed for this node; from ~50 to 99 MYA [21,65-70]. Therefore, we performed the analyses considering three alternative normally distributed calibrations priors for this node: 90 MYA (Std 6), 75 MYA (Std 6), and 60 (Std 6), which collectively span the entire range of age estimates for the node. We show the chronogram for the intermediate date (75 MYA), but we also report the ages of critical nodes in Additional file 2. The analysis was run twice independently for 20 million generations. The numerical results were visualized and compared using Tracer version 1.5 [71]. We estimated stationarity by examining the trace of likelihood values, which typically stabilized in less than one million generations, and we conservatively discarded 10% (2 million generations) as burnin. In addition to the methods discussed above, we also analysed this divergence using a variety of other calibration strategies and priors and the results were highly consistent (i.e. deepest divergence within South American *Bolitoglossa* is always in the early to Middle Miocene). The purpose of this analysis was to test if divergence time estimates within an endemic South America clade of *Bolitoglossa* are significantly earlier than 3 MYA, which would suggest that *Bolitoglossa* either arrived in South American prior to the Pliocene Panamanian land bridge.

Ancestral areas of *Bolitoglossa* were reconstructed using the likelihood-based dispersal-extinction-cladogenesis analysis with LAGRANGE v. 12120508 [66,72]. The analyses were performed on the species tree chronogram of bolitoglossines based on the 75 MYA calibration. The geographical regions for the terminal taxa were either Central America, South America, or both regions, and we did not constrain dispersal probabilities.

### Phylogenetic analyses

Modeltest version 3.7 [73] implemented with PAUP* [74] was used on each gene and codon position separately and both genes together to infer the best model of molecular evolution, which was selected using the Akaike Information Criterion. Sequence divergences between haplotypes and clades were calculated in MEGA4 [75] with a K2P correction [76] for *Cytb* and no correction for *Rag1* (because of its lower mutation rate).

To reconstruct the evolutionary relationships among upper Amazonian and Andean *Bolitoglossa* with all sample and geographic information, Bayesian phylogenetic analyses were run in MrBayes version 3.1.2 [77] under a variety of partition and model settings for each codon position separately and some combined, as calculated from Modeltest (Additional file 7). Prior distributions on state frequencies, substitution rates, gamma distribution shape and the proportion of invariable sites were unlinked across all partitions and left at default values. Branch length prior was left at default for single gene analyses and was set to Unconstrained: Exponential (100) for the combined analysis. Site-specific rates were allowed to vary across partitions (ratepr=variable). Temperature among the MCMC heated chains varied from 0.05 to 0.08 as needed to facilitate chain swapping. The starting tree was random as determined by MrBayes. Each analysis was run in two independent concurrent blocks of 3 million (*Rag1*) or 5 million (*Cytb* and combined) generations with sampling every 100 generations. The first 10 000 trees (*Rag1*) or 15,000 trees (*Cytb* and combined) were discarded as burn-in. Convergence was assumed when chain swaps ranged between 0.4 and 0.8, harmonic means were effectively identical from run 1 and run 2, and parameter PSRF values were approximately 1.00. The partitioning, parameters and model chosen for the final tree were those that provided the highest Bayes factor compared to other models, except if the second best model differed in Bayes factor by less than 10 and was simpler [78]. Fifty percent majority consensus trees were built from the first two independent runs and visualized in FigTree version 1.3.1 (http://tree.bio.ed.ac.uk/).

## Competing interests

The authors declare that they have no competing interests.

## Authors’ contributions

KRE carried out new specimen sampling, sequencing, and molecular genetic analyses, and drafted the manuscript. RMB did sequencing and divergence time and ancestral area analyses. DBW participated in study design, interpretation, and taxonomic issues. SCL participated in study design and interpretation. All authors contributed to writing the manuscript. All authors approved the final manuscript.

## Supplementary Material

Additional file 1 **Bayesian chronogram with 95% highest posterior density for Central and South American *Bolitoglossa *based on *Rag1* and *Cytb.*** Click here for file

Additional file 2 Divergence time estimates based on alternative calibrations. Click here for file

Additional file 3 **Inter-individual K2P corrected sequence divergence at cytochrome b. ***Sensu lato* interspecific comparisons are shaded grey. Click here for file

Additional file 4 **Inter-individual uncorrected sequence divergence at *Rag1.****Sensu lato* interspecific comparisons are shaded grey. Click here for file

Additional file 5 Multilocus combined phylogeny. Click here for file

Additional file 6 List of specimens (species, GenBank accession, continent) included in the divergence time analysis. Click here for file

Additional file 7 Best likelihood models of sequence evolution for each codon position and combined positions. Click here for file
