# Supplementary material for: Early Miocene origin and cryptic diversification of South American salamanders
Source: BMC Evol Biol. 2013 Mar 4;13:59. doi: 10.1186/1471-2148-13-59 (PMC3602097; doi:10.1186/1471-2148-13-59)

Additional file 1 –Bayesian chronogram with 95% highest posterior density for Central and South American *Bolitoglossa* based on *Rag1* and *Cytb*.

The numbers subtending each node are posterior probabilities of the relationships. The bars are the height of the 95% highest posterior density (HPD), which are shown for branches with posterior probabilities greater than 0.50. The analysis was performed in Beast and based on calibrating the base of the crown group Plethodontidae at 75 MYA (6 Std).

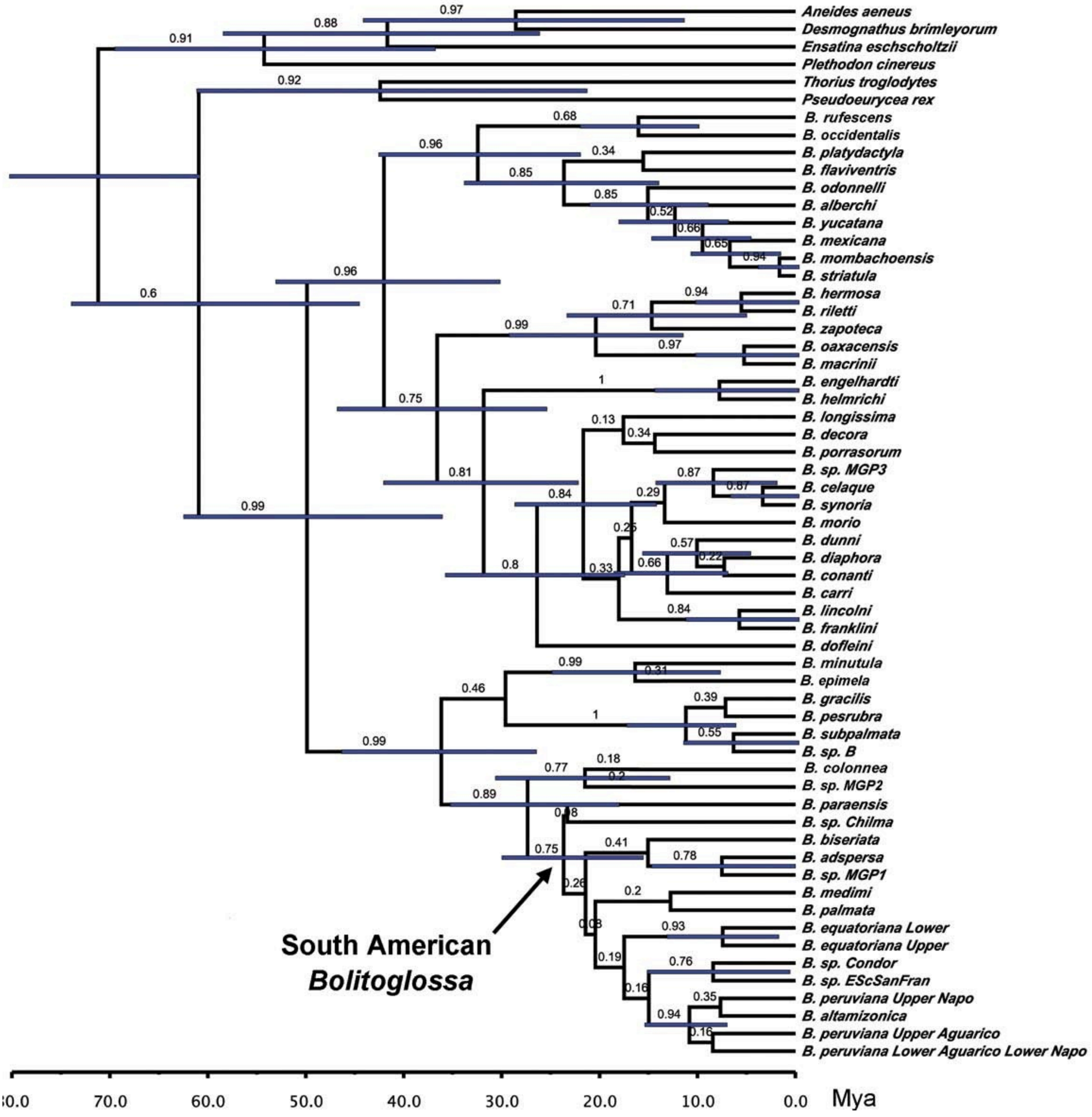

Supplement: Additional file 1 — Bayesian chronogram with 95% highest posterior density for Central and South American Bolitoglossa based on Rag1 and Cytb. [file 1471-2148-13-59-S1.pdf]
