# Supplementary material for: Early Miocene origin and cryptic diversification of South American salamanders
Source: BMC Evol Biol. 2013 Mar 4;13:59. doi: 10.1186/1471-2148-13-59 (PMC3602097; doi:10.1186/1471-2148-13-59)
Supplement: Additional file 2 — Divergence time estimates based on alternative calibrations. [file 1471-2148-13-59-S2.pdf]

Additional file 2 – Divergence time estimates based on alternative calibrations.

Alternative calibrations of 60 (std 6) and 90 (std 6) million years for the crown group Plethodontidae. The calibration date of 75 (std 6) million years was used in the results of Figure 2. SA = South American, Std = standard deviation, HPD = highest posterior density.

	**Norm 60 (Std 6)**	**Norm 60 (Std 6)**	**Norm 60 (Std 6)**	**Norm 75 (Std 6)**	**Norm 75 (Std 6)**	**Norm 75 (Std 6)**	**Norm 90 (Std 6)**	**Norm 90 (Std 6)**	**Norm 90 (Std 6)**
Clade	Average	Min 95% HPD	Max 95% HPD	Average	Min 95% HPD	Max 95% HPD	Average	Min 95% HPD	Max 95% HPD
Bolitoglossa	40.2	29.1	50.2	50.3	36.4	62.8	60.4	43.7	75.4
Eladinea	29.2	21.4	37.4	36.5	26.8	46.7	43.8	32.2	56.0
**SA *Bolitoglossa***	18.9	12.7	24.2	23.6	15.9	30.3	28.3	19.1	36.4
***B. peruviana* (*sensu lato*)**	8.6	5.5	12.3	10.8	6.9	15.4	13.0	8.3	18.5
***B. equatoriana* (*sensu lato*)**	5.9	0.4	12.1	7.4	0.5	15.1	8.9	0.6	18.1
