# Supplementary material for: Early Miocene origin and cryptic diversification of South American salamanders
Source: BMC Evol Biol. 2013 Mar 4;13:59. doi: 10.1186/1471-2148-13-59 (PMC3602097; doi:10.1186/1471-2148-13-59)
Supplement: Additional file 4 — Inter-individual uncorrected sequence divergence at Rag1.Sensu lato interspecific comparisons are shaded grey. [file 1471-2148-13-59-S4.pdf]

Additional file 4 – Inter-individual uncorrected sequence divergence at *Rag1*. *Sensu lato* interspecific comparisons are shaded grey.

			1	2	3	4	5	6	7	8	9	10	11	12	13	14	15	16	17	18
	1	QCAZ25522 Kapawi cf peruviana																		
"altamazonica"	2	QCAZ32291 Yasuni sp	0.022																	
	3	QCAZ20845 PUCE equatoriana	0.010	0.016																
upper Napo peruviana	4	QCAZ25320 Cando cf peruviana	0.010	0.017	0.006															
	5	QCAZ25753 EBJSIV cf peruviana	0.014	0.015	0.005	0.006														
	6	QCAZ25387 LaSelva cf peruviana	0.009	0.022	0.010	0.009	0.014													
	7	QCAZ25385 LaSelva cf peruviana	0.014	0.025	0.011	0.010	0.015	0.002												
lower Napo/lower	8	QCAZ28404 cf peruviana	0.020	0.029	0.010	0.013	0.016	0.015	0.016											
Aguarico peruviana	9	QCAZ25592 Auca cf peruviana	0.014	0.020	0.003	0.009	0.010	0.014	0.015	0.015										
	10	QCAZ25268 Auca cf peruviana	0.011	0.018	0.001	0.008	0.008	0.013	0.014	0.013	0.000									
	11	QCAZ25593 Auca cf peruviana	0.017	0.026	0.009	0.014	0.016	0.019	0.021	0.023	0.011	0.001								
Agua Rico peruviana	12	QCAZ25794 AguaRico cf peruviana	0.027	0.036	0.018	0.026	0.028	0.029	0.029	0.034	0.018	0.015	0.025							
upper equatoriana	13	QCAZ25448 EBJS equatoriana	0.024	0.034	0.014	0.022	0.021	0.025	0.027	0.028	0.018	0.015	0.024	0.032						
equatoriana sensu stricto	14	QCAZ37304 BU0230 Tiputini equatoriana	0.018	0.025	0.005	0.014	0.013	0.018	0.019	0.019	0.010	0.006	0.015	0.025	0.008					
	15	QCAZPaul ECSanFran sp	0.016	0.024	0.004	0.011	0.011	0.015	0.016	0.016	0.009	0.005	0.013	0.025	0.016	0.007				
	16	QCAZ39981 Chilma sp	0.029	0.034	0.014	0.024	0.024	0.028	0.028	0.027	0.019	0.017	0.026	0.035	0.026	0.020	0.016			
	17	QCAZ41724 Condor sp	0.015	0.021	0.003	0.011	0.009	0.015	0.016	0.015	0.008	0.004	0.011	0.023	0.014	0.005	0.000	0.018		
	18	biseriata	0.036	0.041	0.021	0.031	0.031	0.034	0.037	0.034	0.029	0.025	0.031	0.044	0.037	0.029	0.024	0.026	0.025	
	19	MVZ233516 cerroensis	0.037	0.047	0.025	0.036	0.035	0.039	0.041	0.041	0.031	0.025	0.032	0.045	0.034	0.030	0.029	0.029	0.026	0.032
