# Supplementary material for: Early Miocene origin and cryptic diversification of South American salamanders
Source: BMC Evol Biol. 2013 Mar 4;13:59. doi: 10.1186/1471-2148-13-59 (PMC3602097; doi:10.1186/1471-2148-13-59)

Additional file 5 - Multilocus combined phylogeny

The 50% majority-rule phylogenetic tree inferred from combined Bayesian analyses of *Cytb* and *Rag1* sequence data. Posterior probabilities are located at nodes or above branches. Clades referred to in the text are noted in vertical. See Table 1 and Figure 1 for more information on sample localities.

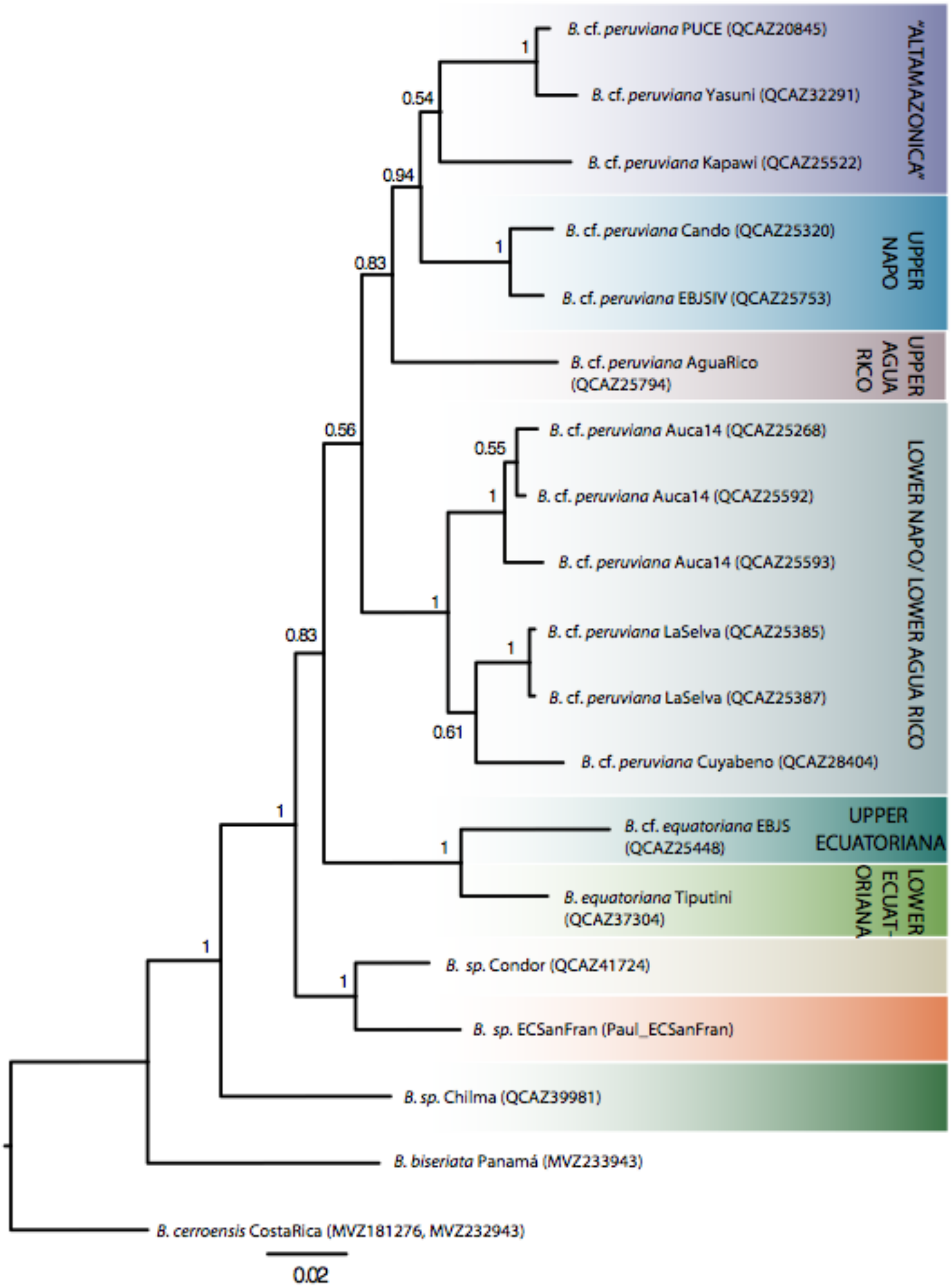

Supplement: Additional file 5 — Multilocus combined phylogeny. [file 1471-2148-13-59-S5.pdf]
