# Supplementary material for: Early Miocene origin and cryptic diversification of South American salamanders
Source: BMC Evol Biol. 2013 Mar 4;13:59. doi: 10.1186/1471-2148-13-59 (PMC3602097; doi:10.1186/1471-2148-13-59)
Supplement: Additional file 6 — List of specimens (species, GenBank accession, continent) included in the divergence time analysis. [file 1471-2148-13-59-S6.pdf]

Additional file 6 – List of specimens (species, GenBank accession, continent) included in the divergence time species tree analysis (x = included) and/or the pruned ancestral area reconstruction, which included fewer representatives per clade.

Genus	species (and clade)	Cytb	Rag1	Continent	Species tree	Ancestral area
Aneides	*aeneus*	AY691742	AY691701	North America	x	x
Desmognathus	*brimleyorum*	AY691737	AY691697	North America	x	x
Ensatina	*eschscholtzii*	AY691743	AY691702	North America	x	x
Plethodon	*cinereus*	AY691745	AY691703	North America	x	x
Pseudoeurycea	*rex*	AY691757	AY650125	Central America	x	x
Thorius	*troglodytes*	KC614433	KC614460	Central America	x	x
Bolitoglossa	*adspersa*	AF212984		South America	x	x
Bolitoglossa	*alberchi*	AF212978		Central America	x	
Bolitoglossa	*alberchi*	AF212979		Central America	x	x
Bolitoglossa	*altamazonica*	AY526160		South America	x	
Bolitoglossa	*biseriata*	AY526161	KC614436	Central America	x	x
Bolitoglossa	*carri*		KC614458	Central America	x	x
Bolitoglossa	*carri*	AY526175		Central America	x	
Bolitoglossa	*carri*	AY526176		Central America	x	
Bolitoglossa	*celaque*	AY526177		Central America	x	x
Bolitoglossa	*celaque*	AY526178		Central America	x	
Bolitoglossa	cf. *equatoriana* (upper equatoriana clade)	DQ353842	KC614451	South America	x	x
Bolitoglossa	cf. *equatoriana* (upper equatoriana clade)	DQ353840		South America	x	
Bolitoglossa	cf. *equatoriana* (upper equatoriana clade)	DQ353841		South America	x	
Bolitoglossa	cf. *equatoriana* (upper equatoriana clade)	DQ353843		South America	x	
Bolitoglossa	cf. *equatoriana* (upper equatoriana clade)	DQ353844		South America	x	
Bolitoglossa	cf. *equatoriana* (upper equatoriana clade)	DQ353846		South America	x	
Bolitoglossa	cf. *peruviana (*altamazonica clade)	DQ353809	KC614442	South America	x	x
Bolitoglossa	cf. *peruviana (*altamazonica clade)	KC614427	KC614453	South America	x	x
Bolitoglossa	cf. *peruviana (*altamazonica clade)	KC614430	KC614455	South America	x	x
Bolitoglossa	cf. *peruviana (*altamazonica clade)	DQ353810		South America	x	
Bolitoglossa	cf. *peruviana (*altamazonica clade)	DQ353811		South America	x	
Bolitoglossa	cf. *peruviana* (lower Napo/lower Aguarico clade)	DQ353836	KC614450	South America	x	x
Bolitoglossa	cf. *peruviana* (lower Napo/lower Aguarico clade)	DQ353830		South America	x	
Bolitoglossa	cf. *peruviana* (lower Napo/lower Aguarico clade)	DQ353831		South America	x	

Bolitoglossa	cf. *peruviana* (lower Napo/lower Aguarico clade)	DQ353832		South America	x	
Bolitoglossa	cf. *peruviana* (lower Napo/lower Aguarico clade)	DQ353833		South America	x	
Bolitoglossa	cf. *peruviana* (lower Napo/lower Aguarico clade)	DQ353834		South America	x	
Bolitoglossa	cf. *peruviana* (lower Napo/lower Aguarico clade)	DQ353835		South America	x	
Bolitoglossa	cf. *peruviana* (lower Napo/lower Aguarico clade)	DQ353837		South America	x	
Bolitoglossa	cf. *peruviana* (lower Napo/lower Aguarico clade)	DQ353839		South America	x	
Bolitoglossa	cf. *peruviana* (lower Napo/lower Aguarico clade)	KC614454		South America	x	
Bolitoglossa	cf. *peruviana* (lower Napo/lower Aguarico clade)	KC614444		South America		x
Bolitoglossa	cf. *peruviana* (upper Aguarico clade)	DQ353815	KC614443	South America	x	x
Bolitoglossa	cf. *peruviana* (upper Aguarico clade)	DQ353812		South America	x	
Bolitoglossa	cf. *peruviana* (upper Aguarico clade)	DQ353813		South America	x	
Bolitoglossa	cf. *peruviana* (upper Aguarico clade)	DQ353814		South America	x	
Bolitoglossa	cf. *peruviana* (upper Napo clade)	DQ353821	KC614445	South America	x	x
Bolitoglossa	cf. *peruviana* (upper Napo clade)	DQ353827	KC614446	South America	x	x
Bolitoglossa	cf. *peruviana* (upper Napo clade)	DQ353816		South America	x	
Bolitoglossa	cf. *peruviana* (upper Napo clade)	DQ353817		South America	x	
Bolitoglossa	cf. *peruviana* (upper Napo clade)	DQ353818		South America	x	
Bolitoglossa	cf. *peruviana* (upper Napo clade)	DQ353820		South America	x	
Bolitoglossa	cf. *peruviana* (upper Napo clade)	DQ353822		South America	x	
Bolitoglossa	cf. *peruviana* (upper Napo clade)	DQ353823		South America	x	
Bolitoglossa	cf. *peruviana* (upper Napo clade)	DQ353824		South America	x	
Bolitoglossa	cf. *peruviana* (upper Napo clade)	DQ353825		South America	x	
Bolitoglossa	cf. *peruviana* (upper Napo clade)	DQ353826		South America	x	
Bolitoglossa	cf. *peruviana* (upper Napo clade)	DQ353828		South America	x	
Bolitoglossa	cf. *peruviana* (upper Napo clade)	DQ353829		South America	x	
Bolitoglossa	*colonnea*	AY526162		Central America	x	x
Bolitoglossa	*conanti*	AY526179		Central America	x	x
Bolitoglossa	*conanti*		KC699924	Central America	x	x
Bolitoglossa	*decora*	AY526180		Central America	x	x
Bolitoglossa	*diaphora*	AY526181		Central America	x	x
Bolitoglossa	*dofleini*	AF212988		Central America	x	x
Bolitoglossa	*dunni*	AY526182	KC614438	Central America	x	x
Bolitoglossa	*engelhardti*	AF212987		Central America	x	x
Bolitoglossa	*engelhardti*		KC699925	Central America	x	x
Bolitoglossa	*epimela*	AF212097		Central America	x	x
Bolitoglossa	*equatoriana*	DQ353845		South America	x	
Bolitoglossa	*equatoriana*	KC614428		South America	x	x

Bolitoglossa	*equatoriana* (was *peruviana* in Parra-Olea et al 2004)	AY526169		South America	x	
Bolitoglossa	*flaviventris*	AF212983		Central America	x	x
Bolitoglossa	*franklini*	AY526184	KC614439	Central America	x	x
Bolitoglossa	*gracilis*	AF212067		Central America	x	x
Bolitoglossa	*gracilis*	AF212068		Central America	x	
Bolitoglossa	*helmrichi*	AY691755	AY650124	Central America	x	x
Bolitoglossa	*hermosa*	AF416678		Central America	x	x
Bolitoglossa	*lincolni*	AY526185	KC614440	Central America	x	x
Bolitoglossa	*longissima*	AY526186	KC614441	Central America	x	x
Bolitoglossa	*macrinii*	AF416680		Central America	x	x
Bolitoglossa	*medemi*	AY526163	KC614437	Central America	x	x
Bolitoglossa	*mexicana*	AF212099		Central America	x	
Bolitoglossa	*mexicana*	AF212975		Central America	x	
Bolitoglossa	*mexicana*	AF212976		Central America	x	
Bolitoglossa	*minutula*	AF212098	KC614434	Central America	x	x
Bolitoglossa	*mombachoensis*	AY133486		Central America	x	x
Bolitoglossa	*morio*		KC699926	Central America	x	x
Bolitoglossa	*morio*	AF212986		Central America	x	
Bolitoglossa	*morio*	AY526187		Central America	x	
Bolitoglossa	*oaxacensis*	AF416681		Central America	x	x
Bolitoglossa	*occidentalis*	AY526158	KC614435	Central America	x	x
Bolitoglossa	*odonnelli*	AF212977	KC699922	Central America	x	x
Bolitoglossa	*palmata*	AY526164		South America	x	x
Bolitoglossa	*palmata*	AY526165		South America	x	
Bolitoglossa	*paraensis*	AY526166		South America	x	x
Bolitoglossa	*paraensis*	AY526167		South America	x	
Bolitoglossa	*paraensis*	AY526168		South America	x	
Bolitoglossa	*peruviana*	AY526170		South America	x	
Bolitoglossa	*pesrubra*	AF212069		Central America	x	x
Bolitoglossa	*platydactyla*	AF212981	KC699923	Central America	x	x
Bolitoglossa	*platydactyla*	AY133484		Central America	x	
Bolitoglossa	*porrasorum*	AY526188		Central America	x	x
Bolitoglossa	*riletti*	AF416682		Central America	x	x
Bolitoglossa	*rufescens*	AY526159		Central America	x	x
Bolitoglossa	*schizodactyla*	AY526171		Central America	x	x
Bolitoglossa	*sp.* (Chilma)	KC614431	KC614456	South America	x	x
Bolitoglossa	*sp.* (Condor)	KC614432		South America	x	x

Bolitoglossa	*sp.* (ECSanFran)	KC699921	South America	x	x
Bolitoglossa	*sp.B*	AF212088	Central America	x	x
Bolitoglossa	*sp.MGP-1*	AY526173	South America	x	x
Bolitoglossa	*sp.MGP-2*	AY526174	Central America	x	x
Bolitoglossa	*sp.MGP-3*	AY526191	Central America	x	x
Bolitoglossa	*sp.MGP-3*	AY526192	Central America	x	x
Bolitoglossa	*subpalmata*	AF212094	Central America	x	x
Bolitoglossa	*synoria*	AY526193	Central America	x	x
Bolitoglossa	*yucatana*	AF212980	Central America	x	x
Bolitoglossa	*zapoteca*	AF416683	Central America	x	x
Bolitoglossa	*zapoteca*	AF416684	Central America	x	
