# Supplementary material for: Early Miocene origin and cryptic diversification of South American salamanders
Source: BMC Evol Biol. 2013 Mar 4;13:59. doi: 10.1186/1471-2148-13-59 (PMC3602097; doi:10.1186/1471-2148-13-59)
Supplement: Additional file 7 — Best likelihood models of sequence evolution for each codon position and combined positions. [file 1471-2148-13-59-S7.pdf]

Additional file 7 - Best likelihood models of sequence evolution for each codon position and combined positions.

Models were selected by AIC score and were used to inform the likelihood parameters of the MrBayes phylogenetic analysis. For the Bayesian phylogenetic analyses, the best model for *Cytb* was with position 1, 2 and 3 separate. The best model for *Rag1* was also with position 1, 2 and 3 separate.

	position 1	position 2	position 1+2 combined	position 3	position 1+2+3 combined
cyt b	HKY+I	TVM+I	TrN+I+G	SYM+G	HKY+I+G
rag1	TVM	F81+I	TVM+I	TVM+G	TVMef+I+G
